# Effect of Lymphatic Filariasis and Hookworm Infection on Pregnancy Course and Outcome in Women Living in the Democratic Republic of the Congo

**DOI:** 10.4269/ajtmh.20-1422

**Published:** 2021-05-03

**Authors:** Jérémy T. Campillo, Emmanuel B. Chabot, Naomi-Pitchouna Awaca-Uvon, Jean-Paul Tambwe, Godefroy Kuyangisa-Simuna, Michel Boussinesq, Cédric B. Chesnais, Sébastien D. Pion

**Affiliations:** 1 UMI 233, Institut de Recherche pour le Développement (IRD), INSERM Unité 1175 and University of Montpellier, Montpellier, France;; 2 UMR1027, Institut National de la Santé et de la Recherche Nationale (Inserm) and University of Toulouse, Toulouse, France;; 3 Programme National de Lutte contre les Maladies Tropicales Négligées à Chimiothérapie Préventive, Ministère de la Santé Publique, Kinshasa, Democratic Republic of the Congo

## Abstract

Little is known about the effect of helminth infections on the natural gynecological and pregnancy course. Our goal was to assess the relationship between *Wuchereria bancrofti* and hookworm (HW) infections with pregnancy course and outcome in a group of 82 women living in a rural area of the Democratic Republic of the Congo. Demographics and information on gynecological and obstetrical histories were collected retrospectively with standardized questionnaires. *Wuchereria bancrofti* and HW infections were diagnosed using a filarial antigen-detection test and the Kato–Katz method, respectively. Analyses consisted of multivariable logistic regressions adjusting for age, number of deliveries, and history of anthelmintic treatment (HAHT). The median age of study participants was 35 (interquartile range [IQR]: 30–44) years, and the median number of deliveries was five (IQR: 3–7). *Wuchereria bancrofti* and HW infection rates were 44.5% and 43.3%, respectively. Filarial antigenemia and HW infection were not significantly associated with the number of deliveries. The proportions of women with a history of pregnancy resulting in neonatal death, miscarriage, premature birth, and postpartum hemorrhage were 56%, 44%, 23%, and 36%, respectively. History of pregnancy associated with neonatal death was less frequent in women with HAHT, tended to be more frequent in women with filarial antigenemia, and was not associated with HW infection. None of the three other pregnancy events studied (miscarriage, premature birth, and postpartum hemorrhage) were associated with filarial antigenemia or HW infection. The positive association found between HAHT and lower risk of neonatal death warrants investigation in larger groups of women.

## INTRODUCTION

Many infectious diseases can cause infertility in males or females, as well as lead to adverse pregnancy outcomes.^1–3^ Infertility, defined as “a disease of the reproductive system defined by the failure to achieve a clinical pregnancy after 12 months or more of regular unprotected sexual intercourse,”^4^ can arise because of “male factors” (such as alterations in sperm concentration and/or motility and/or morphology), female factors, or both. The most common causes of female infertility are ovulatory disorders, tubal occlusion or abnormalities, pelvic adhesions, and endometriosis. Sexually transmitted infections with bacteria *Chlamydia trachomatis* and *Neisseria gonorrhoeae* are a major cause of endometritis and of salpingitis, both of which can result in tubal occlusion and pelvic adhesions, leading to infertility.^5^ Protozoan infections have also been reported as causes of male and female infertility due to local inflammatory processes or hormonal disorders arising from infection.^6^ Evidence also supports potential impacts of helminthic parasites on fertility: infection with *Schistosoma haematobium* can cause tubal occlusion, leading to infertility and ectopic pregnancy,^7–10^ and infection with *Schistosoma* sp. can also induce hormonal imbalances and dysregulation associated with infertility.^10^ Infertility was found to be significantly associated with residence in areas of high *S. haematobium* prevalence in East Africa.^11^ Adult stages of the filarial worm *Wuchereria bancrofti* (the main cause of lymphatic filariasis [LF]) have been found in nodules or lymphatics of the genital tract, where they have been shown to lead to salpingitis, blockage of the fallopian tubes, and ectopic pregnancy.^12–15^ Microfilariae of *W. bancrofti* and other filarial species such as *Loa loa* and *Mansonella perstans* have been found in the follicular fluid and in cervicovaginal smears, but the impact of this presence on fertility is uncertain.^16–19^ Results of a single community-wide study suggest that LF infection has no effect on fertility, despite a strong positive association being found between being microfilaremic and abnormal menstruation patterns in women aged ≥ 30 years.^20^ It has also been suggested that *L. loa* and *M. perstans* microfilaremia may affect the functions of the hormonal system, leading to delays in puberty, disturbances to the menstrual cycle, and infertility in older subjects.^21^ Soil-transmitted helminths (STH) might also have an impact on fertility. In a longitudinal study conducted in Bolivia, infection with *Ascaris lumbricoides* was found to be associated with earlier first births and shortened inter-birth intervals, whereas infection with hookworm (HW) was associated with delayed first pregnancy and extended inter-birth intervals.^22^ Whether this impact of STH on fertility is due to immunological phenomena is a matter of debate.^23^ The worms *Enterobius vermicularis* and, to a lesser extent, *A. lumbricoides* can induce tubo-ovarian lesions, leading to infertility or ectopic pregnancies,^24,25^ but these cases are exceptional, and STH do not seem to have a significant impact on the reproductive potential before the implantation of the embryo in the uterus.

Viral, bacterial, and parasitic infections can also impact the pregnancy outcomes, by increasing the risk of abortion, congenital anomalies, stillbirth, intrauterine growth retardation, preterm birth, or neonatal death.^26–31^ Besides the ectopic pregnancies described earlier, the effects of maternal infection with *Schistosoma* sp. on the pregnancy outcomes are not well known.^32–35^ The same uncertainties exist regarding the effect of maternal filarial infections. In some studies, risk of abortion and/or postnatal deaths was higher in females with filariasis than that in controls, or higher in areas where LF was highly endemic than in non-endemic areas.^36,37^ Evidence is mixed however; in a study conducted in Sri Lanka, neither gestation age at delivery nor birthweight differed significantly between females with or without circulating filarial antigens (CFAs).^38^ The effects of maternal infection with *Schistosoma* sp. or STH on the pregnancy outcomes have been assessed by several cross-sectional or longitudinal studies, including clinical trials with anthelmintic drugs. Because of extensive variability in study designs, outcomes assessed, and levels of infection across studies, as well as numerous potential confounding factors, the effects of *Schistosoma* and STH on pregnancy outcomes remain unclear.^39–42^ In this context, we conducted a retrospective study in two villages in the Democratic Republic of the Congo (DRC) to evaluate the effect of LF and HW (*Necator americanus* or *Ancylostoma duodenale*) on pregnancy outcomes in female residents.

## METHODS

### Study population and selection of subjects.

The study was conducted in June–July 2018 in two neighboring villages (Misai and Mbumkimi) located in the Kwilu Province of the DRC. These villages had been previously selected for a community trial which started in 2014 and whose objective was to assess the impact of community biannual treatment with albendazole alone on LF and STH infections.^43,44^ During this trial, all individuals aged ≥ 2 years were offered treatment with a single dose of albendazole (400 mg) every 6 months, and volunteers aged ≥ 5 years had yearly parasitological assessment for LF and STH infections. For the purpose of the present study, we invited women aged at least 15 years in 2018 to answer a standardized questionnaire including sociodemographic and obstetric history information. This questionnaire was applied by the nurse in charge of these two villages. Consenting women were included only if they had been married for at least 2 years.

### Obstetric history and outcomes.

The following information was collected from surveyed women: number of deliveries, number of living children, number of dead children, number of miscarriages, number of children born before 8 months of pregnancy (preterm birth), number of newborns who died within 1 month of delivery (neonatal mortality), and number of postpartum hemorrhages (see questionnaire in Supplementary Material). Statistical analyses focused on four pregnancy adverse outcomes derived from these surveys: history of miscarriage, history of preterm birth, history of neonatal mortality, and history of postpartum hemorrhage, all represented as binary (presence/absence) variables. We also assessed the relationship between the number of deliveries and the infection (either LF or HW) status of the participants.

### Parasitological status regarding LF and STH.

From 2014, all participants in the albendazole trial were invited to undergo a rapid test assessing their LF infection status. This test was delivered before their first treatment and then annually thereafter. This was performed using the Filarial Test Strip (FTS) which detects CFAs in the blood (indicative of the presence of live adult worms).^45^ The test was used according to the manufacturer’s instructions (Alere, Scarborough, ME). Most participants in the present study had received their first albendazole treatment in 2014 and were followed up until the date of the present study in 2018. The first FTS result obtained during the trial was used in the analyses (as a proxy for chronic LF infection) to define LF-infected versus LF-non-infected women. The use of a recent result (at the time of inclusion in the albendazole trial) as a proxy for the historical level of infection in the participants is relevant because longitudinal studies (up to 26 years of follow-up) have shown that, in the absence of treatment, individual status regarding *W. bancrofti* remains fairly stable over time.^46–49^

Hookworm infection in study participants was diagnosed by the examination of stool samples using the double-slide Kato–Katz method. In a manner similar to LF, we considered that individual HW infection status remains relatively stable over time in the absence of treatment. This assumption is based on the fact that 1) studies comparing egg densities before and after treatment in the same individuals suggest a predisposition to infection (and thus chronically exposed to a given level of exposure. Hence, the result of the earliest particularly in women)^50–54^ and 2) intensity of exposure to HW is closely related to environmental factors,^51,55^ and one can assume that, once married, females living in rural African areas remain more or less in the same perimeters of activities; stool examination performed during the course of the trial was used in the analyses presented here.

### Other independent variables.

Before inclusion in the albendazole community trial, participants were asked whether they had ever taken anthelminthic drugs before commencement of the trial in 2014. In the statistical analysis, we used the history of anthelmintic treatment (HAHT) before 2014 as a binary variable (yes versus no). Participants were grouped into four balanced age categories (< 31, 31–35, 36–44, and ≥ 45 years), with the number of deliveries analyzed using four categories for the bivariate analysis (< 4, 4–5, 6–7, and ≥ 8), and two categories for the logistic regression (< 5 versus ≥ 5) because of the small sample size.

### Statistical analysis.

Each independent quantitative variable was described by its arithmetic mean, SD, median, and interquartile range. Each independent qualitative variable was described using percentages (of total number of women). Bivariate analyses relating LF and HW infection status to demographic and reproductive variables were performed by chi^2^ or Fisher’s exact test if chi^2^ conditions were not respected. For quantitative variables, nonparametric mean comparisons (Mann–Whitney test) were performed. Qualitative ordinal variables were analyzed using Cuzick’s test for trend. In a second step, multivariable logistic regression was used to assess the association between dependent and independent variables while adjusting for potential confounders. Four dependent variables were analyzed using logistic regressions: postpartum hemorrhage, miscarriage, neonatal mortality, and preterm birth, expressed as binary variable (history of adverse outcome or no history of adverse outcome). For logistic regression, we included all variables, whether significant or not; that is, we used saturated models. Some of the questionnaires were not fully completed by the nurse in charge of its application, resulting in missing data. For all models, the significance of relevant interaction terms was assessed (age and infection status, age and HAHT, infection status, and HAHT). For variables with missing data included in the logistic regression, we created a “missing data” category. All statistical analyses were performed using STATA 15.1 (StatCorps, College Station, TX).

## RESULTS

### Study population.

A total of 215 women participated in the parasitological examinations conducted as part of the albendazole trial in 2018, and 113 (52.5%) were at least 15 years old. Of the 113 eligible women, 12 (10.6%) women refused to participate in the study. Thirteen were excluded because they were single or had lived as a couple for less than 2 years. Six others were excluded from the analysis because of ambiguity in the responses or because the questionnaires contained only sociodemographic information. A total of 82 women were thus included in the analysis. Only two of the participants have had no children. Seventy-two of them (87.8%) had participated in the first albendazole distribution in 2014 and were followed up until 2018. Ten others were included in the cohort during subsequent years (five in 2015, three in 2016, and two in 2018). Table 1 summarizes the information regarding the sociodemography and the variables related to reproductive health in the study population. The mean age of the participants was 37 years (range: 17–74). By comparison to the 82 women included in the analyses, those 13 who have been excluded were significantly younger (mean age: 25 years, *P* < 0.001) but did not differ in terms of HW or LF infection prevalence (*P* = 0.135 and 0.770, respectively).

**Table 1:** Description of main sociodemographic variables

Variable	
Age (years)	
Mean (SD)	37 (11.7)
Median (IQR)	35 [30–44]
Already gave birth, *n* (%)	
Yes	80 (97.6)
No	2 (2.4)
Number of deliveries	
Mean (SD)	5.2 (2.6)
Median (IQR)	5 [3–7]
Currently pregnant, *n* (%)	
Yes	10 (16.4)
No	51 (83.6)
History of postpartum hemorrhage	
Yes	21 (36.2)
No	37 (63.8)
Number of postpartum hemorrhage	
Mean (SD)	1.15 (2.23)
Median (IQR)	2 [1–5]
History of miscarriage, *n* (%)	
Yes	28 (43.7)
No	36 (56.3)
Number of miscarriages	
Mean (SD)	0,8 (1.2)
Median (IQR)	1 [1–2]
History of neonatal mortality, *n* (%)	
Yes	45 (56.2)
No	35 (43.8)
History of preterm birth	
Yes	12 (22.6)
No	41 (77.4)
Number of preterm births	
Mean (SD)	0.32 (0.75)
Median (IQR)	1 [1–1]
History of anthelminthic treatment before the first albendazole treatment given as part of the trial, *n* (%)	
Yes	51 (62.2)
No	31 (37.8)

IQR = interquartile range. For reproductive health variables, means were calculated on all the participants and medians only on those who experienced the event.

### Parasitological characteristics and bivariate analyses.

Thirty-four of the 82 women included in the analyses (44.5%) were positive by the FTS test before their first albendazole treatment. Only 60 (45 in 2014 and 15 in 2015) women provided a stool sample for examination; HW infection rate was 43.3% (26/60). Table 2 shows the main reproductive health variables considered here, stratified according to the participants’ LF infection status. The presence of filarial antigenemia was not associated with the history of miscarriage (*P* = 0.847), history of preterm birth (*P* = 0.730), history of postpartum hemorrhage (*P* = 0.587), history of neonatal mortality (*P* = 0.264), or with the number of deliveries (*P* = 0.772). Table 3 shows the main reproductive health variables, stratified according to HW infection status. Hookworm infection was not associated with the history of miscarriage (*P* = 1.000), history of premature birth (*P* = 0.670), history of postpartum hemorrhage (*P* = 0.294), history of neonatal mortality (*P* = 0.531), or with the number of deliveries (*P* = 0.396). The number of deliveries (expressed as a continuous variable) was not associated with HW infection (5.54 deliveries in infected women versus 4.60 in noninfected, in mean; *P* = 0.141, Mann–Whitney test) nor LF infection (5.72 deliveries in infected women versus 4.97 in noninfected, in mean; *P* = 0.158, Mann–Whitney test).

**Table 2:** Distribution of sociodemographic and reproductive health variables according to the FTS result (presence or absence of *Wuchereria bancrofti*)

Variable	Total	FTS-	FTS+	*P*-value
Age-group (years), *n* (%)				0.111
< 31	22 (26.8)	11 (50.0)	11 (50.0)	
31–35	20 (24.4)	16 (80.0)	4 (20.0)	
36–44	20 (24.4)	12 (60.0)	8 (40.0)	
≥ 45	20 (24.4)	9 (45.0)	11 (55.0)	
Age (continuous)				0.394
Mean (SD)	35.30 (12.49)	33.98 (11.02)	37.12 (14.21)	
Current pregnancy, *n* (%)				0.524
Yes	10 (16.4)	6 (60.0)	4 (40.0)	
No	51 (83.6)	28 (54.9)	23 (45.1)	
MD	21	14	7	
First trimester miscarriage, *n* (%)				0.574
Yes	14 (28.0)	9 (64.3)	5 (35.7)	
No	36 (72.0)	20 (55.6)	16 (44.4)	
MD	32	19	13	
Miscarriage, *n* (%)				0.847
Yes	28 (43.7)	17 (60.7)	11 (39.3)	
No	36 (56.3)	21 (58.3)	15 (41.7)	
MD	18	10	8	
Preterm birth, *n* (%)				0.730
Yes	12 (58.3)	7 (58.3)	5 (41.7)	
No	41 (77.4)	28 (68.3)	13 (31.7)	
MD	29	13	16	
Postpartum hemorrhage, *n* (%)				0.587
Yes	21 (36.2)	14 (66.7)	7 (33.3)	
No	37 (63.8)	22 (59.5)	15 (40.5)	
MD	24	12	12	
Neonatal mortality, *n* (%)				0.264
Yes	45 (56.2)	24 (53.3)	21 (46.7)	
No	35 (43.8)	23 (65.7)	12 (34.3)	
MD	2	1	1	
Number of deliveries (categories), *n* (%)				0.772
< 4	24 (30.0)	15 (62.5)	9 (37.5)	
4–5	19 (23.7)	12 (63.2)	7 (36.8)	
6–7	22 (27.5)	13 (59.1)	9 (40.9)	
≥ 8	15 (18.8)	7 (46.7)	8 (53.3)	
MD	2	1	1	
Number of deliveries (continuous)				
Mean (SD)	5.29 (2.60)	4.97 (2.61)	5.72 (2.56)	0.158

FTS = Filarial Test Strip; MD = missing data. For binary outcomes, the chi^2^ test has been used (or Fisher’s exact test if chi^2^ conditions were not respected). For quantitative variables, the Mann–Whitney test has been performed.

**Table 3:** Distribution of sociodemographic and reproductive health variables according to the presence or absence of HW at the Kato–Katz stool examination

Variable	Total	HW-	HW+	*P*-value
Age-group (years), *n* (%)				0.098
< 31	19 (31.7)	11 (57.9)	8 (42.1)	
31–35	13 (21.7)	9 (69.2)	4 (30.8)	
36–44	14 (23.3)	10 (71.4)	4 (28.6)	
≥ 45	14 (23.3)	4 (28.6)	10 (71.4)	
Age (continuous)				
Mean (SD)	34.91 (13.11)	33.14 (10.57)	36.91 (15.42)	0.461
Current pregnancy, *n* (%)				1
Yes	8 (18.6)	4 (50.0)	4 (50.0)	
No	35 (81.4)	20 (57.1)	15 (42.9)	
MD	17	10	7	
First trimester miscarriage, *n* (%)				1
Yes	7 (21.2)	5 (71.4)	2 (28.6)	
No	26 (78.2)	16 (61.5)	10 (38.5)	
MD	27	13	14	
Miscarriage, *n* (%)				1
Yes	13 (30.9)	8 (61.5)	5 (38.5)	
No	29 (69.1)	19 (65.5)	10 (34.5)	
MD	18	7	11	
Preterm birth, *n* (%)				0.670
Yes	6 (16.2)	3 (50.0)	3 (50.0)	
No	31 (83.8)	19 (61.3)	12 (38.7)	
MD	23	12	11	
Postpartum hemorrhage, *n* (%)				0.294
Yes	12 (31.6)	9 (75.0)	3 (25.0)	
No	26 (68.4)	14 (53.8)	12 (46.2)	
MD	22	11	11	
Neonatal mortality, *n* (%)				0.531
Yes	31 (53.5)	17 (54.8)	14 (45.2)	
No	27 (46.5)	17 (63.0)	10 (37.0)	
MD	2	0	2	
Number of deliveries (categories), *n* (%)				0.396
< 4	18 (31.0)	13 (72.2)	5 (27.8)	
4–5	16 (27.6)	9 (56.3)	7 (43.7)	
6–7	16 (27.6)	8 (50.0)	8 (50.0)	
≥ 8	8 (13.8)	3 (37.5)	5 (62.5)	
MD	2	1	1	
Number of deliveries (continuous), *n* (%)				
Mean (SD)	5.00 (2.44)	4.60 (2.45)	5.54 (2.38)	0. 141

MD = missing data; HW = hookworm.

For binary outcomes, the chi^2^ test has been used (or Fisher’s exact test if chi^2^ conditions were not respected). For quantitative variables, the Mann–Whitney test has been performed.

### Multivariable logistic analyses among pregnancy adverse outcomes, LF and HW infections, and history of anthelminthic treatment.

The results of multivariable logistic regressions to assess the effect of LF and HW infections on the incidence of four pregnancy adverse outcomes are summarized in Table 4. History of neonatal mortality was more frequent in women with a positive FTS, but this was not significant (*P* = 0.135). Hookworm infection had no impact on any of the four main outcomes. Increase in age was strongly correlated with increase in miscarriage and neonatal mortality histories. Age was not included in the preterm birth model because of convergence issues. The number of deliveries was correlated with prematurity (adjusted odds ratio (aOR) = 11.6, *P* = 0.008 for women who had more than five deliveries with less than five deliveries as the reference category). Neonatal mortality was significantly less frequent in women with a HAHT (aOR = 0.2 with 95% CI = [0.04–0.82], *P* = 0.024).

**Table 4:** Logistic regression analysis on the four dependent variables: miscarriage, preterm birth, neonatal mortality, and postpartum hemorrhage

	(1)		(2)		(3)		(4)	
	Miscarriage		Preterm birth		Neonatal mortality		Postpartum hemorrhage	
	OR (95% CI)	*P*-value	OR (95% CI)	*P*-value	OR (95% CI)	*P*-value	OR (95% CI)	*P*-value
*Wuchereria bancrofti*								
Absence	Ref.		Ref.		Ref.		Ref.	
Presence	0.7 (0.2–2.7)	0.562	1.7 (0.3–8.9)	0.544	2.8 (0.7–10.7)	0.135	0.4 (0.1–1.6)	0.187
Hookworm								
Absence	Ref.		Ref.		Ref.		Ref.	
Presence	0.7 (0.1–3.4)	0.612	1.4 (0.2–11.0)	0.739	1.3 (0.3–5.4)	0.698	0.3 (0.1–1.8)	0.199
Missing data*	4.2 (0.9–19.1)	0.061	4.9 (0.6–38.9)	0.134	1.5 (0.3–6.8)	0.606	1.6 (0.4–6.9)	0.494
Age-group (years)			Not included†					
< 30	Ref.				Ref.		Ref.	
30–35	1.6 (0.2–11.0)	0.622			16.2 (2.4–108.3)	0.004	0.3 (0.0–1.7)	0.165
36–44	3.9 (0.5–28.9)	0.185			29.9 (3.2–281.7)	0.003	0.4 (0.0–3.0)	0.350
≥ 45	14.9 (1.5–150.8)	0.022			24.2 (2.5–233.4)	0.006	0.5 (0.1–5.0)	0.591
Number of deliveries								
< 5	Ref.		Ref.		Ref.		Ref.	
≥ 5	1.0 (0.2–4.2)	0.976	11.6 (1.9–70.5)	0.008	1.8 (0.4–7.8)	0.437	2.6 (0.5–13.5)	0.250
History of anthelmintic treatment								
No	Ref.		Ref.		Ref.		Ref.	
Yes	0.4 (0.1–2.0)	0.285	0.1 (0.01–1.5)	0.106	0.2 (0.0–0.8)	0.024	0.9 (0.2–3.7)	0.894
Missing data*	0.9 (0.1–5.5)	0.883	0.5 (0.1–4.7)	0.555	0.3 (0.1–2.1)	0.253	0.3 (0.1–2.0)	0.226
Number of observations‡	64		53		79		58	

OR = odds ratio; Ref. = reference category.

* Missing data were included in the regression model as a categorical variable to improve convergence and number of participants.

† Age could not be included in prematurity logistic regression because of convergence issue due to the relatively small number of participants.

‡ Number of observations are smaller than 82 because of missing information in the questionnaires administrated to women or missing data in parasitological results.

## DISCUSSION

The aim of the present study was to document, for the first time, pregnancy outcomes in a rural population of DRC, and assess whether pregnancy outcomes were related to HW or LF parasitological status. No significant associations were found between infections with *W. bancrofti* or with HW, and the four pregnancy outcomes we focused on (miscarriage, preterm birth, neonatal mortality, and postpartum hemorrhage). The only significant result was that women with HAHT before the first albendazole treatment given as part of the trial had a significantly lower frequency of history of neonatal mortality.

The significant relationship observed here between HAHT and neonatal mortality is intriguing but will require further exploration to confirm and establish whether it is due to biological causes such as anemia (see in the following text) or is instead a product of other factors such as sociological determinants. Despite the potential benefits of anthelmintic treatment among pregnant women and an informal consultation made by the WHO^56^ which recommended treatment of all pregnant women with praziquantel and albendazole in areas endemic for STH or schistosomiasis, widespread adoption has not been observed. To date, only a minority of countries have included anthelmintic intake (albendazole or mebendazole) during routine pregnancy care, in the second and third trimesters of pregnancy: specifically, Madagascar, Nepal, and Sri Lanka.^57^ Published studies suggest that it may be because of a fear of adverse birth outcomes due to a lack of safety data. More studies are needed to assess the benefits of implementing systematic deworming for pregnant women in resource-poor settings.

One of the main limitations of this study is the lack of power because of the small number of women recruited and the missing data in the questionnaires which were frequently incorrectly completed. An additional limitation is that pregnancy outcomes were analyzed using infection as a binary indicator (presence/absence) rather than a continuous one (based on the intensity of infection measured by, e.g., the FTS score for LF or egg counts per gram of feces for HW). This stratification was undertaken because of limitations in the sample size.

Another important limitation of our study is that we did not collect hemoglobin levels from the participating women. Indeed, it is well established that HW infection can cause anemia.^58^ As well, pregnant women are more prone to anemia because of the physiological changes that typically accompany pregnancy.^59^ It is likely then that pregnant women infected with HW are a population at high risk for anemia. Anemia has been implicated in the occurrence of adverse pregnancy events such as perinatal and neonatal mortality,^60^ prematurity, and low birth weight.^61^ Indeed, work from Mpairewe et al.^39^ has shown that infection with helminths such as HW are associated with anemia in pregnant women, and that the risk of anemia was highly correlated with the infection intensity. A final limitation is that we collected retrospective data, and therefore, these data are subject to recall bias.

Our study was a retrospective observational study, but to assess more specifically the relationships between parasitic infections and pregnancy outcomes, prospective cohort studies are required. Examples of such studies are present in the literature: Christian et al.^62^ conducted a clinical trial of albendazole (zero, one, or two doses) among 4,998 pregnant women in Nepal coinfected by the three geohelminths (prevalence of HW: 47.5%). They assessed the proportion of severe anemia in the treated (one or two doses) and untreated groups and found that 20% of the women belonging to the latter suffered of severe anemia (hemoglobin < 70 g/L) versus 5% in the treated group. There was also evidence of an impact on infant mortality (i.e., mortality in the 6 months after birth), which was 14% lower in the one-dose group and 41% lower in the two-dose group than those in the untreated group. In a similar clinical trial conducted in 2006 in Peru among 1,042 women in the second trimester of pregnancy and coinfected by the three geohelminths, Larocque et al.^63^ compared the proportion of anemia in a group treated with placebo and iron supplementation and a group treated with mebendazole (500 mg) and iron supplementation. These authors did not observe a significant difference in anemia proportion at the third trimester, but this could be due to the iron supplementation provided to both groups. However, the study did show that the proportion of very low weight birth infants was higher in the placebo group than that in the mebendazole group.^64^ Although the results appear to be suggestive of impacts of geohelminths on adverse pregnancy outcomes, a limitation of both the studies described earlier is that the effect of STH infection on pregnancy outcome was assessed indirectly, through administration of anthelminthic treatment, rather than directly. A number of other parasites sensitive to the same treatments (*Taenia* spp., *E. vermicularis*, *Strongyloides stercoralis* …) and which frequently co-occur with STHs could therefore be implicated. To our knowledge, the only prospective cohort study exploring the direct implication of HW on the pregnancy outcomes is a Bolivian longitudinal 9-year study conducted in an area with high prevalence of HW. It showed that HW infection was associated with lower body mass index of the women, lower number of deliveries, and an older age at first pregnancy.^22^ Some cross-sectional studies have examined the occurrence of adverse pregnancy events according to the HW infection status. Two of the four published studies on the relationship between HW and infant mortality found an association,^65,66^ whereas the other two found no association.^67,68^ Results in the literature are similarly mixed for the relationship between HW infection and premature birth; Wanyonyi et al.^66^ and Asundep et al.^65^ found an association, whereas Mahande and Mahande^68^ did not.

For LF infection, despite the fact that we did not find any significant association with pregnancy outcomes, our results suggest that LF may have an implication on the neonatal mortality. Because of the small number of women recruited, this possible association has to be reevaluated within a larger study, and using a more suitable design such as a prospective cohort study with recurrent assessments of infection levels, hemoglobin levels, and adverse pregnancy events. A case report published in 2006 showed that an LF infection in a 36-year-old woman may have led to implantation failure in in vitro fertilization cycles.^69^ If LF infection can cause implantation issue during in vitro fertilization cycles, one cannot exclude that the parasite may cause pregnancy outcome issues and fertility issues.

This study describes the frequency of adverse pregnancy events and LF and HW infections in a rural population of the DRC. Although no significant associations were found between infections with *W. bancrofti* or with HW, and miscarriage, preterm birth, neonatal mortality and postpartum hemorrhage, women with HAHT had significantly less frequent history of neonatal mortality. This topic of research is under-explored, and more studies are needed to understand whether HW and LF are involved in the occurrence of adverse pregnancy events.

## Supplemental information

Supplemental materials

## References

[1] Spingarn CL Edelman MH, 1965. Parasitic diseases in relation to pregnancy. Medical, Surgical, and Gynecologic Complications of Pregnancy. Baltmore, MD: The Williams & Wilkins Company. 692–719.

[2] Mascie-Taylor C, 1996. The relationship between disease and subfecundity. Variability in Human Fertility. Cambridge, UK: Cambridge University Press, Vol. 53, 1043.

[3] Pellati D Mylonakis I Bertoloni G Fiore C Andrisani A Ambrosini G Armanini D, 2008. Genital tract infections and infertility. Eur J Obstet Gynecol Reprod Biol 140: 3–11. 18456385 10.1016/j.ejogrb.2008.03.009

[4] Zegers-Hochschild F Adamson GD de Mouzon J Ishihara O Mansour R Nygren K Sullivan EVS, 2009. International committee for monitoring assisted reproductive technology (ICMART) and the world health organization (WHO) revised glossary of ART terminology. Fertil Steril 92: 1520–1524. 19828144 10.1016/j.fertnstert.2009.09.009

[5] Novy MJ Eschenbach DA Witkin SS, 2008. Infections as a cause of infertility. Glob Libr Women’s Med 2008: 1756–2228.

[6] Nourollahpour Shiadeh M Niyyati M Fallahi S Rostami A, 2015. Human parasitic protozoan infection to infertility: a systematic review. Parasitol Res 115: 469–477. 26573517 10.1007/s00436-015-4827-y

[7] Helling-Giese G Kjetland EF Gundersen SG Poggensee G Richter J Krantz I Feldmeier H, 1996. Schistosomiasis in women : manifestations in the upper reproductive tract. Acta Trop 62: 225–238. 9028408 10.1016/s0001-706x(96)00025-3

[8] Poggensee G Feldmeier H Krantz I, 1999. Schistosomiasis of the female genital tract: public health aspects. Parasitol Today 15: 378–381. 10461167 10.1016/s0169-4758(99)01497-0

[9] Kjetland EF Kurewa EN Mduluza T Midzi N Gomo E Friis H Gundersen SG Ndhlovu PD, 2010. The first community-based report on the effect of genital *Schistosoma haematobium* infection on female fertility. Fertil Steril 94: 1551–1553. 20149365 10.1016/j.fertnstert.2009.12.050

[10] Ribeiro AR Luis C Fernandes R Botelho MC, 2019. Schistosomiasis and infertility: what do we know? Trends Parasitol 35: 964–971. 31623951 10.1016/j.pt.2019.09.001

[11] Woodall PA Kramer MR, 2018. Schistosomiasis and infertility in east africa. Am J Trop Med Hyg 98: 1137–1144. 29313478 10.4269/ajtmh.17-0280 PMC5928810

[12] Carayon A Brenot G Camain R Bilharziennes LESL, 1967. 20 lésions tubo-ovariennes de la bilharziose et de la filariose de Bancroft. Bull Soc Méd Afr Noire Lgue Fr 12: 464–473. 5598035

[13] Goel P Tandon R Saha P Prabhakar S Goel B Kaur R Kaur N Singhal N, 2013. A rare case of ovarian and pelvic filariasis. Trop Doct 43: 108–109. 23820743 10.1177/0049475513495021

[14] Panditi S Shelke A Thummalakunta L, 2016. “Filarial dance sign” real-time ultrasound diagnosis of filarial oophoritis. J Clin Ultrasound 44: 500–501. 27130361 10.1002/jcu.22359

[15] Mondal S Adhikari A Chakraborty R Mandal S, 2017. Ovarian filariasis presenting as tubo-ovarian mass: report of a rare case. CHRISMED J Heal Res 4: 136–138.

[16] Wisanto A Laureys M Camus M Devroey P Verheyen G Van Steirteghem AC, 1993. Case report: loa loa microfilariae aspirated during oocyte retrieval. Hum Reprod 8: 2096–2097. 8150909 10.1093/oxfordjournals.humrep.a137988

[17] Chang LW Reller ME Bishop JA Talaat K Nutman TB Auwaerter PG, 2008. A 41‐year‐old woman from Cameroon with infertility. Clin Infect Dis 47: 141–143. 18522508 10.1086/588787 PMC3398835

[18] Brezina P Yunus F Garcia J Zhao Y, 2011. Description of the parasite *Wucheria bancrofti* microfilariae identified in follicular fluid following transvaginal oocyte retrieval. J Assist Reprod Genet 28: 433–436. 21287402 10.1007/s10815-011-9538-4 PMC3151358

[19] Puy V 2017. Detection of *Loa loa* microfilariae in follicular fluid during assisted reproductive technology: case report and review of the literature. Open Forum Infect Dis 4: ofx208. 29670930 10.1093/ofid/ofx208 PMC5903404

[20] Bernhard P Makunde RW Magnussen P Lemnge MM, 2000. Genital manifestations and reproductive health in female residents of a *Wuchereria bancrofti*-endemic area in Tanzania. Trans R Soc Trop Med Hyg 94: 409–412. 11127246 10.1016/s0035-9203(00)90123-8

[21] Mavoungou D Poaty-Mavoungou V Ongali B Akoume MY Maka G Mavoungou E, 2005. Hypothalamic-pituitary gonadal axis and immune response imbalance during chronic filarial infections. Trop Med Int Heal 10: 1180–1186. 10.1111/j.1365-3156.2005.01499.x 16262744

[22] Blackwell AD Tamayo M Beheim B Trumble BC Stieglitz J Hooper PL Martin M Kaplan H Gurven M, 2015. Helminth infection, fecundity, and age of first pregnancy in human females. Science 350: 970–972. 26586763 10.1126/science.aac7902 PMC5953513

[23] Persson G Ekmann JR Hviid TVF, 2019. Reflections upon immunological mechanisms involved in fertility, pregnancy and parasite infections. J Reprod Immunol 136: 102610. 31479960 10.1016/j.jri.2019.08.001

[24] Sterling R Guay A, 1936. Invasion of the female generative tract by *Ascaris lumbricoides*. J AMA 107: 2046–2047.

[25] Brooks TJ Goetz CC Plauché WC, 1962. Pelvic granuloma due to *Enterobius vermicularis*. J Am Med Assoc 179: 492–494. 10.1001/jama.1962.03050070014003 13873477

[26] Reinhardt MC, 1979. Effects of parasitic infections in pregnant women. Ciba Found Symp 77: 149–170. 10.1002/9780470720608.ch10 121975

[27] McFalls JA McFalls MH, 1984. Disease and Fertility. Academic Press Inc. Harcour and Brace Jovanouiclr.

[28] Goldenberg RL Andrews WW Yuan AC Mackay HT Louis S, 1997. Sexually transmitted diseases and adverse outcomes of pregnancy. Clin Perinatol 24: 23–41. 9099500

[29] Goldenberg RL Culhane JF Johnson DC, 2005. Maternal infection and adverse fetal and neonatal outcomes. Clin Perinatol 32: 523–559. 16085019 10.1016/j.clp.2005.04.006 PMC7119141

[30] Coonrod DV 2008. The clinical content of preconception care: infectious diseases in preconception care. Am J Obstet Gynecol 199 (6 Suppl 2): S296–S309. 19081424 10.1016/j.ajog.2008.08.062

[31] Maldonado YA Nizet V Klein JO Remington JS Wilson CB, 2011. Current concepts of infections of the fetus and newborn infant. Infectious Diseases of the Fetus and Newborn Infant. Elsevier Saunders, 3–25.

[32] Friedman JF Mital P Kanzaria HK Olds GR Kurtis JD, 2007. Schistosomiasis and pregnancy. Trends Parasitol 23: 159–164. 17336160 10.1016/j.pt.2007.02.006

[33] Mombo-Ngoma G 2017. Urogenital schistosomiasis during pregnancy is associated with low birth weight delivery: analysis of a prospective cohort of pregnant women and their offspring in Gabon. Int J Parasitol 47: 69–74. 28003151 10.1016/j.ijpara.2016.11.001

[34] Freer JB Bourke CD Durhuus GH Kjetland EF Prendergast AJ, 2018. Schistosomiasis in the first 1000 days. Lancet Infect Dis 18: e193–e203. 29170089 10.1016/S1473-3099(17)30490-5

[35] Murenjekwa W 2019. Determinants of urogenital schistosomiasis among pregnant women and its association with pregnancy outcomes, neonatal deaths, and child growth. *J Infect Dis jiz664*: 1–12. 10.1093/infdis/jiz664 PMC8064048 31832636

[36] Ayres M Salzano FM Helena M Franco LP de Souza Barros RM, 1976. The association of blood groups, ABH secretion, haptoglobins and hemoglobins with filariasis. Hum Hered 26: 105–109. 950235 10.1159/000152789

[37] Jordan P, 1955. Bancroftian filariasis : an assessment of its economic importance in tanganyika. Trans R Soc Trop Med Hyg 49: 271–279. 14396931 10.1016/0035-9203(55)90070-5

[38] Weerasinghe CR De Silva NR Michael E, 2005. Maternal filarial-infection status and its consequences on pregnancy and the newborn, in Ragama, Sri Lanka. Ann Trop Med Parasitol 99: 813–816. 16297296 10.1179/136485905X65198

[39] Mpairwe H Tweyongyere R Elliott A, 2014. Pregnancy and helminth infections. Parasite Immunol 36: 328–337. 24471654 10.1111/pim.12101 PMC4260141

[40] Blackwell AD, 2016. Helminth infection during pregnancy: insights from evolutionary ecology. Int J Womens Health 8: 651–661. 27956844 10.2147/IJWH.S103529 PMC5113914

[41] Campbell SJ Nery SV Doi SA Gray DJ Soares Magalhães RJ McCarthy JS Traub RJ Andrews RM Clements ACA, 2016. Complexities and perplexities: a critical appraisal of the evidence for soil-transmitted helminth infection-related morbidity. PLoS Negl Trop Dis 10: e0004566. 27196100 10.1371/journal.pntd.0004566 PMC4873196

[42] Salam RA Cousens S Welch V Gaffey M Middleton P Makrides M Arora P Bhutta ZA, 2019. Mass deworming for soil-transmitted helminths and schistosomiasis among pregnant women: a systematic review and individual participant data meta-analysis. Campbell Syst Rev 15: 1–27. 10.1002/cl2.1052 PMC8356523 37131518

[43] Chesnais CB Missamou F Pion SD Bopda J Louya F Majewski AC Fischer PU Weil GJ Boussinesq M, 2014. A case study of risk factors for lymphatic filariasis in the Republic of Congo. Parasites and Vectors 7: 300. 24984769 10.1186/1756-3305-7-300 PMC4089930

[44] Pion SDS Chesnais CB Weil GJ Fischer PU Missamou F Boussinesq M, 2017. Effect of 3 years of biannual mass drug administration with albendazole on lymphatic filariasis and soil-transmitted helminth infections: a community-based study in Republic of the Congo. Lancet Infect Dis 17: 763–769. 28372977 10.1016/S1473-3099(17)30175-5

[45] Chesnais CB Vlaminck J Kunyu-Shako B Pion SD Awaca-Uvon NP Weil GJ Mumba D Boussinesq M, 2016. Measurement of circulating filarial antigen levels in human blood with a point-of-care test strip and a portable spectrodensitometer. Am J Trop Med Hyg 94: 1324–1329. 27114288 10.4269/ajtmh.15-0916 PMC4889752

[46] Bloch P Nielsen NO Meyrowitsch DW Malecela MN Simonsen PE, 2011. A 22 year follow-up study on lymphatic filariasis in Tanzania: analysis of immunological responsiveness in relation to long-term infection pattern. Acta Trop 120: 258–267. 21964049 10.1016/j.actatropica.2011.09.006

[47] Meyrowitsch DW Simonsen PE Makunde WH, 1995. A 16 year follow-up study on bancroftian filariasis in three communities of north-eastern Tanzania. Ann Trop Med Parasitol 89: 665–675. 8745941 10.1080/00034983.1995.11813000

[48] Meyrowitsch DW Simonsen PE Magesa SM, 2004. A 26-year follow-up of bancroftian filariasis in two communities in north-eastern Tanzania. Ann Trop Med Parasitol 98: 155–169. 15035726 10.1179/000349804225003172

[49] Sahoo PK Babu Geddam JJ Satapathy AK Mohanty MC Das BK Acharya AS Mishra N Ravindran B, 2002. Bancroftian filariasis: a 13-year follow-up study of asymptomatic microfilariae carriers and endemic normals in Orissa, India. Parasitology 124: 191–201. 11862995 10.1017/s0031182001001007

[50] Breitling LPH Wilson AJ Raiko A Lagog M Siba P Shaw MA Quinnell RJ, 2008. Heritability of human hookworm infection in Papua New Guinea. Parasitology 135: 1407–1415. 18937884 10.1017/S0031182008004976

[51] Quinnell RJ Pullan RL Breitling LP Geiger SM Cundill B Correa-Oliveira R Brooker S Bethony JM, 2010. Genetic and household determinants of predisposition to human hookworm infectionin a Brazilian community. J Infect Dis 202: 954–961. 20681887 10.1086/655813 PMC2938471

[52] Quinnell RJ Griffin J Nowell MA Raiko A Pritchard DI, 2001. Predisposition to hookworm infection in Papua New Guinea. Trans R Soc Trop Med Hyg 95: 139–142. 11355543 10.1016/s0035-9203(01)90138-5

[53] Schad GA Anderson RM, 1985. Predisposition to hookworm infection in humans. Science 228: 1537–1540. 4012307 10.1126/science.4012307

[54] Williams-Blangero S Blangero J Bradley M, 1997. Quantitative genetic analysis of susceptibility to hookworm infection in a population from rural Zimbabwe. Hum Biol 69: 201–208. 9057344

[55] Pullan RL Kabatereine NB Quinnell RJ Brooker S, 2010. Spatial and genetic epidemiology of hookworm in a rural community in Uganda. PLoS Negl Trop Dis 4: e713. 20559556 10.1371/journal.pntd.0000713 PMC2886101

[56] WHO, 1994. Report of the WHO Informal Consultation on Hookworm Infection and Anaemia in Girls and Women. Geneva, Switzerland: World Health Organization.

[57] Brooker S Hotez PJ Bundy DAP, 2008. Hookworm-related anaemia among pregnant women: a systematic review. PLoS Negl Trop Dis 2: e291. 18820740 10.1371/journal.pntd.0000291 PMC2553481

[58] Roche M Layrisse M, 1966. Nature and causes of hookworm anemia. Am J Trop Med Hyg 15: 1029–1102. 5334793

[59] Lindsay HA, 2000. Anemia and iron deficiency: effects on pregnancy outcome. Am J Clin Nutr 7: 1280–1284. 10.1093/ajcn/71.5.1280s 10799402

[60] Steer PJ, 2000. Maternal hemoglobin concentration and birth weight. Am J Clin Nutr 71: 1285–1287. 10.1093/ajcn/71.5.1285s 10799403

[61] McCormick M, 1985. The contribution of low birth weight to infant mortality and childhood morbidity. N Engl J Med 312: 82–90. 3880598 10.1056/NEJM198501103120204

[62] Christian P Khatry SK West KP Jr, 2004. Antenatal anthelmintic treatment, birthweight, and infant survival in rural Nepal. Lancet 364: 981–983. 15364190 10.1016/S0140-6736(04)17023-2

[63] Larocque R Casapia M Gotuzzo E MacLean JD Soto JC Rahme E Gyorkos TW, 2006. A double-blind randomized controlled trial of antenatal mebendazole to reduce low birthweight in a hookworm-endemic area of Peru. Trop Med Int Heal 11: 1485–1495. 10.1111/j.1365-3156.2006.01706.x 17002722

[64] Gyorkos TW Gilbert NL Larocque R Casapía M Montresor A, 2012. Re-visiting *Trichuris trichiura* intensity thresholds based on anemia during pregnancy. PLoS Negl Trop Dis 6: 22–25. 10.1371/journal.pntd.0001783 PMC3441397 23029572

[65] Asundep NN Jolly PE Carson AP Turpin CA Zhang K Wilson NO Stiles JK Tameru B, 2014. Effect of malaria and geohelminth infection on birth outcomes in Kumasi, Ghana. J Trop Dis Heal 4: 582–594. 10.9734/IJTDH/2014/7573 PMC4235765 25414840

[66] Wanyonyi WA Mulambalah CS Mulama DH Omukunda E Siteti DI, 2018. Malaria and geohelminthiasis coinfections in expectant women: effect on maternal health and birth outcomes in a malaria endemic region in Kenya. J Parasitol Res 2018: 2613484. 30631589 10.1155/2018/2613484 PMC6304650

[67] Yatich NJ 2010. The effect of malaria and intestinal helminth coinfection on birth outcomes in Kumasi, Ghana. Am J Trop Med Hyg 82: 28–34. 20064991 10.4269/ajtmh.2010.09-0165 PMC2803505

[68] Mahande AM Mahande MJ, 2016. Prevalence of parasitic infections and associations with pregnancy complications and outcomes in northern Tanzania: a registry-based cross-sectional study. BMC Infect Dis 16: 78. 26874788 10.1186/s12879-016-1413-6 PMC4753041

[69] Bazi T Finan R Zourob D Sabbagh AS Nasnas R Zreik TG, 2006. Filariasis infection is a probable cause of implantation failure in in vitro fertilization cycles. Fertil Steril 85: 1822.e13–1822.e15. 10.1016/j.fertnstert.2005.11.067 16677646

