# Supplementary material for: Effect of Lymphatic Filariasis and Hookworm Infection on Pregnancy Course and Outcome in Women Living in the Democratic Republic of the Congo
Source: Am J Trop Med Hyg. 2021 May 3;104(6):2074–81. doi: 10.4269/ajtmh.20-1422 (PMC8176502; doi:10.4269/ajtmh.20-1422)
Supplement: Supplementary file 1 [file tpmd201422.SD1.pdf]

Are you in a regular relationship?	Yes<br>No
How long have you been in a regular relationship?	__ years
Have you been a regular couple for more than 2 years?	Yes<br>No
Have you ever given birth to a child?	Yes<br>No
How many sons are alive?<br>How many daughters are alive?	_____<br>_____
How many boys have died and how many girls have died?	_____<br>_____
How many boys have died in the month following the birth?<br>How many girls have died in the month following the birth?	_____<br><br>_____
Are you currently pregnant?	
Apart from pregnancies that resulted in live births, do you have another pregnancy that lasted only a few weeks or a few months, or that ended in the birth of a stillborn child?	Yes<br>No
How many times has this happened?	_____
How many times did this happen in the first quarter?	_____
Age at first pregnancy	_____
How many children were born at term (> 8 months)?	_____
How many children were born prematurely (< 8 months)?	_____
Number of hemorrhages from delivery during childbirth?	_____
